# Solid‐state NMR protocols for unveiling dynamics and (drug) interactions of membrane‐bound proteins

**DOI:** 10.1002/pro.70102

**Published:** 2025-03-18

**Authors:** Alessia Lasorsa, Patrick C. A. van der Wel

**Affiliations:** ^1^ Zernike Institute for Advanced Materials University of Groningen Groningen the Netherlands

**Keywords:** drugs, dynamics, membrane proteins, solid‐state NMR, structural biology

## Abstract

Magic angle spinning solid‐state NMR (MAS ssNMR) is a versatile tool for studying the structure and dynamics of membrane proteins, as well as their interactions with ligands and drugs. Its power lies in the ability to provide atomic‐level information on samples under physiological‐like conditions. Moreover, it can illuminate dynamics across a wide range of timescales with great relevance to membrane protein function and dysfunction. In this protocol paper, we highlight key aspects of sample preparation, data acquisition, and interpretation, based on our own experience and the broader literature. We discuss key protocol steps along with important considerations for sample preparation and parameters for ssNMR measurements, with reference to the special requirements of membrane‐based samples. Such samples display physiologically relevant dynamics across different motional regimes that can be probed by NMR but also can interfere with certain NMR measurements. We guide the reader through the whole process from sample preparation to complex NMR characterization techniques. Throughout the report, we refer back to examples from our own prior work on the interactions between cytochrome c and cardiolipin‐containing membranes, with a discussion of the lipid dependence and interactions with a peroxidase‐activity inhibitor. We conclude with a short discussion of alternative and new methods that are further boosting the power and versatility of ssNMR as a tool to study membrane‐bound proteins and their ligands or drug interactions.

## 1 INTRODUCTION

Membrane proteins represent approximately 23% of the human proteome (Uhlén et al. 2015), but they are 60% of current drug targets (Overington et al. 2006; Yin and Flynn 2016). Developing drugs that target membrane proteins requires structural information and techniques to detect and analyze drug–protein interactions. Progress in experimental techniques like cryo‐electron microscopy (cryo‐EM), X‐ray crystallography, and nuclear magnetic resonance (NMR) enables the determination of membrane protein structures. However, there is more to a protein than its structure, and there is a growing interest in understanding the role of dynamics in the function and dysfunction of membrane proteins. For instance, the (allosteric) dynamics of G‐protein‐coupled receptors (GPCRs) are crucial for understanding and modulating their function (Conflitti et al. 2025; Jin et al. 2023).

Here we discuss protocols in modern solid‐state (ss)NMR to probe membrane protein structure and dynamics, with a secondary focus on the relevance of these techniques to drug or ligand interaction studies (Franks et al. 2012; Miao and Cross 2013; Stöppler et al. 2018). One advantage of ssNMR lies in its ability to study proteins associated with a liquid‐crystalline lipid bilayer, which closely mimics the natural membrane environment (Singer and Nicolson 1972). This is crucial as the functional properties of membrane proteins are often (at least in part) determined by interactions with the membrane (McKay et al. 2018). The variety of experiments ssNMR can perform is huge, which highlights a large versatility but also implies, at times, confounding complexity. By necessity, this protocol paper will focus on select experiment types, without being able to cover all possible approaches offered by modern ssNMR.

**FIGURE 1:**
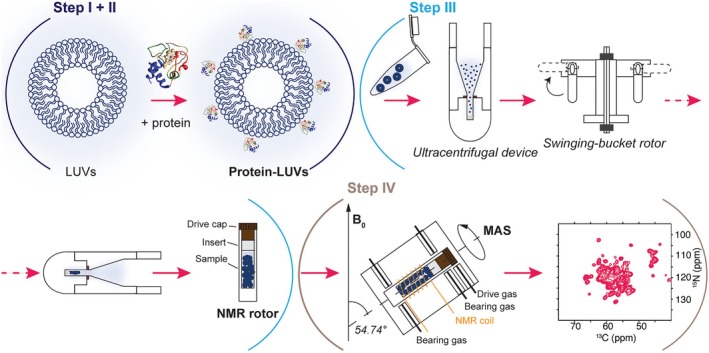
Schematic representation of key steps in ssNMR studies of protein–lipid complexes. Steps I–II (see text) involve protein production (with isotope labeling) and protein–membrane complex formation. Step III is the transfer of the protein–lipid complex into the ssNMR rotor (using a centrifugal device). Step IV is the acquisition of MAS ssNMR spectra and their analysis and interpretation.

In general, the process of studying lipid–protein complexes using ssNMR can be summarized in four steps (Figure 1): (I) protein production and purification, (II) protein reconstitution into lipid vesicles, (III) transfer of the semisolid material in the ssNMR rotor, and (IV) data collection and interpretation (Figure 1). We will outline each of these stages, using as an example a lipid–protein complex that we have being studying: the cytochrome c (cyt c)‐lipid complex (Li et al. 2019; Li et al. 2021; Mandal et al. 2015). The interaction of cyt c with cardiolipin (CL) lipids is a pivotal step in mitochondrial apoptosis, and a pathogenic event in, for example, Barth syndrome (Kagan et al. 2005; Kagan et al. 2023). The interaction of cyt c with specific lipid types modifies the protein's structure, dynamics, and function. In particular, it can trigger a “gain of function,” in which cyt c becomes a potent lipid peroxidase that generates CL derivatives that act as down‐stream triggers of apoptosis. Molecules that modulate or inhibit this gain‐of‐function are of interest as potential drugs for various diseases (Kagan et al. 2023). We will take a step by step look at our typical experimental approach, with additional discussion of select other literature. Details of sample preparation and spectroscopy can be found in the cited papers, with the current report focused on the overall approach taken.

Our main focus is on the use of magic‐angle‐spinning (MAS) NMR, in which whole‐sample rotation relative to the imposed magnetic field is used to obtain high‐quality NMR spectra. This is the most widely used type of ssNMR at this time and permits studies of numerous kinds of solid or semi‐solid samples. A complementary approach for membrane protein ssNMR is the use of aligned or oriented samples, which do not have to be spun in the magnetic field (Aisenbrey et al. 2013; Hansen et al. 2015). Oriented ssNMR is a powerful method but less widely used and not a focus of the current report.

## 2 RESULTS AND DISCUSSION

### 2.1 Step I: Preparation of isotopically labeled proteins

In‐depth MAS NMR characterization typically requires proteins that are enriched (labeled) with desired NMR‐sensitive stable isotopes, such as ^13^C and/or ^15^N. The low natural abundance of these isotopes (^13^C: 1.1%; ^15^N: 0.4%) limits the NMR sensitivity in the absence of labeling. Moreover, multidimensional NMR relies on polarization transfer between adjacent active spins (e.g., ^13^C‐^13^C or ^13^C‐^15^N), which is typically not feasible with unlabeled proteins. Therefore, proteins are typically produced with isotope labeling, for which a variety of procedures have been developed. A classic approach is to use bacterial cells (*Escherichia coli*) that can easily be combined with minimal media supplemented with ^13^C and/or ^15^N‐labeled carbon and nitrogen sources. Increasingly, more complex cell types (insect, yeast, mammalian) are being used to produce challenging (human) proteins that are unsuitable for bacterial production (e.g., GPCRs). Aside from uniform labeling of the whole protein, a powerful approach is to apply selective labeling techniques, in which only certain atoms or amino acids are labeled (Li et al. 2021; Skrisovska et al. 2010). This approach is of high relevance to membrane proteins, which may suffer from substantial peak overlap, peak broadening, and low signal intensities (more below). Targeted labeling strategies can simplify spectra and even allow the effective use of 1D spectra to probe amino acids or protein segments of particular relevance (Cady et al. 2010; Li et al. 2021; Spooner and Watts 1992). Deuteration (LeMaster 1989) can be useful to minimize the effect of abundant homonuclear (^1^H‐^1^H) and heteronuclear (^1^H‐^13^C and ^1^H‐^15^N) dipolar interactions, especially in studies that use ^1^H‐detection (typically combined with fast MAS). The purification of the labeled proteins is frequently performed with a chromatography‐based approach, often facilitated by the inclusion of affinity tags. During the purification process, detergents may be essential to avoid misfolding or aggregation of the hydrophobic (integral) membrane proteins (Seddon et al. 2004). This is less of a factor for conditional or peripheral membrane proteins (like cyt c), which are soluble in the absence of specific lipids.

### 2.2 Step II: Reconstitution of membrane–protein complexes

Detergent‐based micelles can stabilize membrane proteins, for example, in solution NMR (Seddon et al. 2004). They offer several advantages, including ease of use and a wide choice of detergent scaffolds. However, small micelles, with favorable (solution) NMR relaxation properties, can lead to incorrect folding or loss of enzymatic activity. To better mimic biological membranes, liposomes such as multilamellar vesicles (MLVs) or large unilamellar vesicles (LUVs; Figure 1) can be used. LUVs are composed of a single spherical lipid bilayer with a defined vesicular diameter (typically several hundred nm), which can be determined via different techniques (Figure S1, Supporting Information). MLVs are larger and are instead composed of multiple concentric lipid bilayers with intervening aqueous layers. Depending on the context, one may opt for one of these membrane‐mimicking systems.

MLVs have the disadvantage that their onion‐like layering (Figure S1 d) differs from natural cellular membranes, which are usually present as single lipid bilayers. The multilayer structure makes the lipid bilayer more ordered and less dynamic. The different (lipid) dynamics of MLVs and LUVs allow them to be distinguished by (static) ^31^P ssNMR measurements (Figure S1 d). The higher order in MLVs can benefit the spectral quality in some canonical ssNMR experiments (relying on dipolar transfer techniques, as discussed below). LUVs are more like cellular membranes as they feature a single lipid bilayer and exhibit more dynamics. However, compared to most cellular membranes, the relatively small size of LUVs implies more curvature than one expects in, for example, the plasma membrane. Moreover, lipid diffusion and vesicular tumbling in smaller LUVs can cause reductions in (dipolar) order parameters and relaxation times, impacting ssNMR line shapes (see also Figure S1 d). Undulations or fluctuations of the bilayer are additional slow‐time‐scale phenomena that can negatively impact NMR relaxation properties. In extreme cases, these membrane dynamics can prevent high‐quality ssNMR spectra from being acquired. Thus, based on ssNMR considerations alone, the membrane ordering effect of MLVs may be preferred.

Yet, there are cases where biological relevance or experimental considerations favor LUVs over MLVs. The abovementioned cyt c protein binds to membrane surfaces, making MLVs less attractive (given the burial of membrane surfaces within the multi‐layered vesicles). Thus, for many of our studies, we prepared LUVs using a mix of lipids that mimics the relevant cellular membrane (van Meer et al. 2008). Here, this was a mitochondrial membrane, characterized by the presence of special cardiolipin (CL) lipids. The employed phospholipids were obtained in purified form from commercial suppliers. They are first mixed in the desired composition in chloroform, then the organic solvent is removed by drying under nitrogen gas and then under vacuum overnight. The dried film of mixed lipids is hydrated with buffer, suspended, and subjected to freeze–thaw cycles to generate a homogeneously mixed hydrated lipid bilayer. Typically, such a process yields MLVs. To create LUVs, the sample is forced several times through a special porous membrane with 100–400 nm pores (above the lipid melting temperature). This extrusion process is a common method to obtain unilamellar vesicles homogenous in size and shape, with their diameter determined by the pore size of the filter used. Post‐extrusion, the actual size can be verified by dynamic light scattering, small‐angle X‐ray scattering, or electron microscopy (Figure S1).

In the case of a peripherally bound protein like cyt c, one can simply add solutions of the (labeled) protein to such pre‐formed protein‐free membranes. For integral membrane proteins, more complex procedures are often necessary, commonly with a dialysis‐based step that safely guides a detergent‐stabilized protein complex into a membrane‐embedded state. In either case, a resulting protein–membrane complex is now ready for MAS NMR analysis. Crucial parameters to report (and optimize) are the choice of lipid composition and the protein/lipid molar ratio. Both modulate the behavior of the lipid membrane and associated proteins, and thus need to be carefully considered. Biological relevance may be optimal for low protein: lipid ratios, but ssNMR sensitivity benefits from higher ratios. Practical studies tend to employ ratios (P:L) ranging from 1:20 to 1:100, with lower values resulting in overly low protein signals. Even higher P:L ratios can be achieved in some cases, for example, when 2D crystals are formed (Eddy et al. 2012).

### 2.3 Step III: Transfer of the semisolid material in a ssNMR rotor

The earlier steps should yield a hydrated suspension of protein‐containing liposomes. Due to the limited sensitivity of MAS NMR, one typically tries to limit the excess of hydration water to maximize the amount of protein/lipid in the final sample (Mandal et al. 2017). A sample holder (MAS rotor) in a MAS NMR experiment is commonly a small ceramic tube with an outer diameter between 0.7 and 4 mm, with internal volumes from less than a microliter to several dozens of microliters. This implies that the total sample mass is less than a mg to dozens of mgs, which includes the protein, lipids, and buffer. Biological relevance requires that at least 40%–50% of the sample should consist of aqueous buffer (Bechinger and Seelig 1991; Dvinskikh et al. 2005). An overly large excess of water takes up space not available to the lipid–protein complexes and thus reduces the achievable NMR sensitivity.

There are several ways to prepare samples with controlled, but sufficient hydration. In our group, we heavily rely on a sedimentation‐type approach to turn protein/lipid suspensions into suitable MAS NMR samples. To do so, we pellet the vesicles and pack the sample into the rotor in a single step, using a custom‐built, funnel‐shaped ultracentrifugal device in a swinging bucket ultracentrifuge (Figure 1; step II) (Bertini et al. 2012; Böckmann et al. 2009; Mandal et al. 2017). The required speed of (ultra)centrifugation depends on the sample type. While MLVs are often easier to pellet, LUVs resist sedimentation due to their smaller density and size (Tortorella et al. 1993). The protein–lipid complex is pelleted directly into the MAS rotor, after which excess supernatant is removed. This avoids dehydration of the protein, to optimally preserve its structure and dynamics. However, this approach yields samples that retain a substantial amount of water, and the final amount of water can be difficult to estimate precisely. One may instead dry (or lyophilize) the protein‐containing membranes, and then add an exact amount of water by pipetting or by incubating the dry sample in an atmosphere with controlled high humidity. This (re)hydration process can be done with the (dry) sample in the MAS rotor, or prior to transfer into the MAS rotor with (low/modest speed) centrifugation (Hisao et al. 2016).

Once the sample is packed into the rotor, and any excess material removed, the rotor is sealed with caps that should result in a well‐closed rotor. The proper sealing is essential to avoid sample leakage or spinning instability. At the same time, it is advisable not to pack the sample overly full, to reduce the risks of the cap being dislodged during MAS, which could have disastrous consequences for both sample and probe. One crucial step that we take prior to any ssNMR analysis is to weigh the finished and packed sample rotor. One of the possible concerns during extended NMR measurements is dehydration, which can be verified by comparing the total rotor mass before and after ssNMR analysis.

### 2.4 Step IV: MAS NMR analysis

The next step is the actual ssNMR analysis. We will discuss an approach that centers on ^13^C‐detected MAS NMR at moderate MAS rates, between 8 and 24 kHz MAS. This regime is accessible with MAS rotors of a diameter of 4 mm or less, with 3.2 mm MAS rotors being particularly popular at this time. With smaller rotor sizes, one can reach higher MAS rates, which increasingly enable ^1^H‐detected MAS NMR that can be very powerful for (membrane) protein studies (Medeiros‐Silva et al. 2016; Stöppler et al. 2018). Given space limitations, we will not focus on this approach in the current protocol, noting that analogous considerations described here similarly apply to fast‐MAS ^1^H‐detected approaches. Moreover, we will not discuss standard but crucial preparative experiments such as shimming, magic angle adjustments, power and temperature calibrations, and (external/indirect) chemical shift referencing.

### 2.5 Sample fingerprinting with 1D ^1^H MAS NMR spectroscopy

**FIGURE 2:**
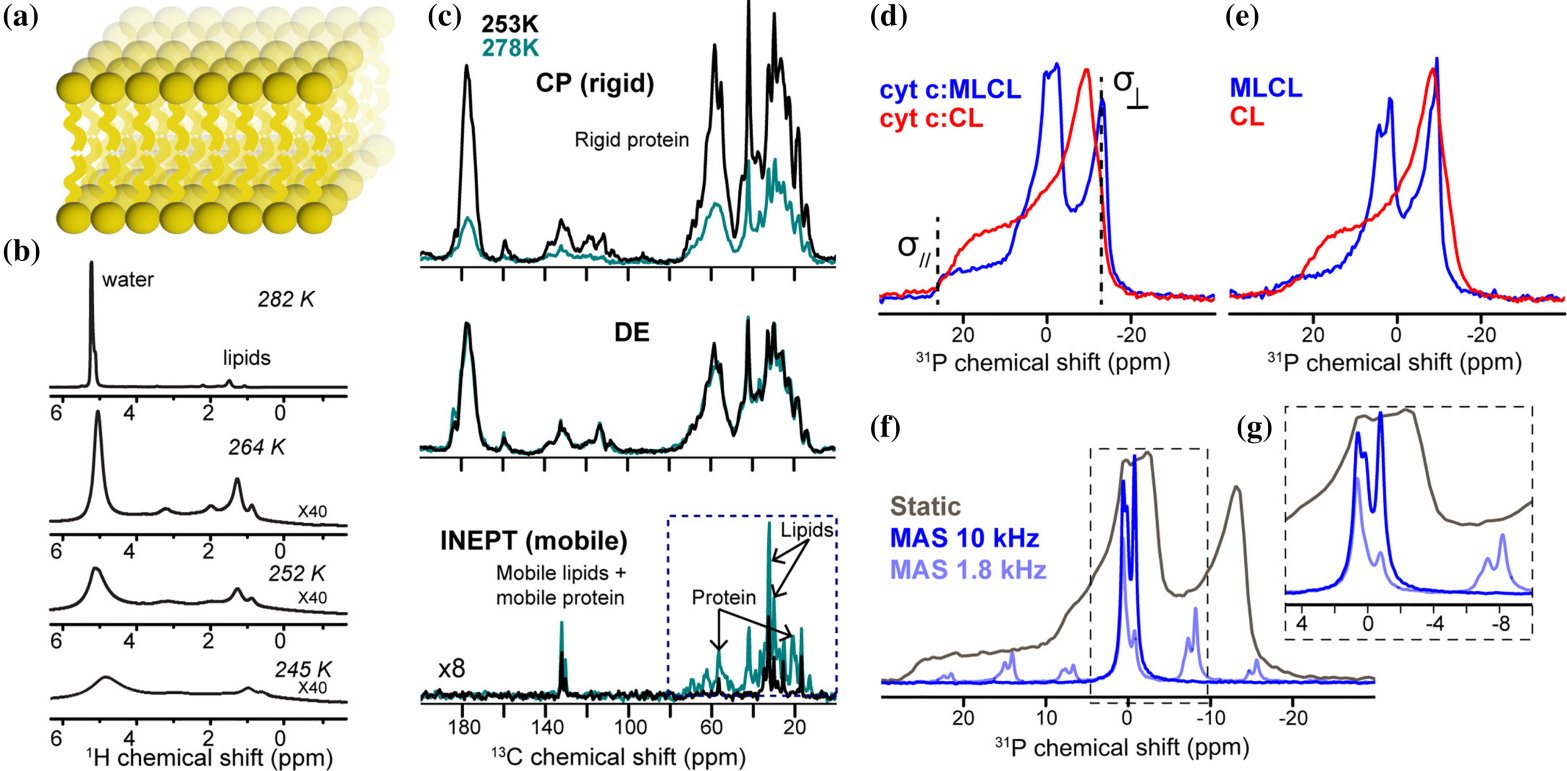
1D MAS NMR spectra of cyt c complexed with CL‐containing membranes. (a) Schematic graphic of lipid bilayer. (b) ^1^H 1D MAS NMR spectra recorded on cyt c bound to CL/PC membranes at different measurement temperatures. Reprinted from Li et al. (2019). (c) ^13^C 1D MAS NMR spectra (using CP, DE, and INEPT) of cyt‐c bound to membranes containing lyso‐CL (MLCL). (d, e) 1D ^31^P ssNMR static spectra of DOPC:MLCL (1:1) (blue) and DOPC:CL (1:1) (red) vesicles without and with bound cyt c. See Figure S2 for reference ^31^P spectra on different types of lipid phases. (f) 1D ^31^P ssNMR MAS spectra of DOPC:MLCL (1:1) at 10 kHz (blue) and 1.8 kHz (light blue) MAS rates, compared to the static spectrum. (g) Zoomed‐in region showing the specific sideband feature (dashed box from (f)). Panels (c–g) reprinted from Kagan et al. (2023) with permission from Springer Nature.

Our first set of experiments on a new sample is typically recording simple but valuable 1D experiments that provide an initial “fingerprinting” and analysis of the sample. As noted above, biological samples are typically studied in hydrated form. Although not always published, we generally start a series of NMR measurements with simple ^1^H 1D spectra (under MAS; Figure 2b). In the absence of homonuclear decoupling, the ^1^H signals from immobile proteins are not usually visible. Instead, the ^1^H spectrum will be dominated by signals from the water in the sample, as well as any (fluid) lipids. This is illustrated in Figure 2b for a sample consisting of membrane lipids with bound cyt c (Li et al. 2019; Mandal et al. 2015). Such data can be used to probe lipid phase behavior or different water pools in the sample (El Hariri Nokab et al. 2022; Mandal et al. 2015; Mandal and van der Wel 2016; Wang et al. 2017). Another feature that may be detected is the presence of (unexpected) small molecule contaminants (or unbound drugs or ligands) in solution. For us, one main reason is to use the initial ^1^H spectrum as a “fingerprint” that allows the detection of sample dehydration (manifesting as a change in the water peak). Moreover, it can reveal (large scale) changes in sample integrity or structure. As such, it is advisable to start a measurement series with such a spectrum, and to also end with it, with the possible inclusion of additional test measurements during an extended measurement series that can last days.

### 2.6 Structural and dynamic fingerprinting with 1D ^13^C MAS NMR spectroscopy

Next, we turn to ^13^C‐detected MAS NMR, again starting with a series of simple 1D spectra. While multidimensional NMR can provide impressive atomic‐detail information, simple 1D spectra are essential tools for rapid sample characterization, fingerprinting, and quality control. Notably, MAS NMR offers multiple different ways of acquiring a ^13^C 1D spectrum, using different types of polarization transfer techniques. Most common are the use of direct ^13^C excitation (DE), ^1^H‐^13^C cross‐polarization (CP) and ^1^H‐^13^C insensitive nuclei enhanced by polarization transfer (INEPT) (Figure 2c). As discussed in many different papers (Matlahov and van der Wel 2018; Nowacka et al. 2010; Warschawski and Devaux 2000), these methods have distinct sensitivities to molecular motion, and thus permit spectral editing or spectral filtering based on dynamics (Matlahov and van der Wel 2018). We refer readers to more in‐depth discussions of the underlying spin physics in several recent papers (Aebischer and Ernst 2024; Topgaard 2023). In simple terms, the efficiency of each technique depends on the presence or absence of motional averaging of dipolar interactions, and the impact of such dynamics on T_1_ and T_2_ relaxation times.

DE experiments should yield spectra that give a (semi) quantitative representation of the relative amounts of molecules in the sample, as long as the recycle delay between scans is sufficiently long. Thus, DE measurements can be used to estimate the relative amounts of different components. Samples (or sample domains) lacking molecular motion can have very long T_1_ values that translate into diminished peak intensities when the recycle delay is kept (too) short. Figure 2c (middle) shows the DE spectra for membranes with bound cyt c, where the protein is uniformly ^13^C‐ and ^15^N‐labeled, but the employed lipids are unlabeled. Since the protein: lipid molar ratio is 1:25, the ^13^C DE spectrum is dominated by the (labeled) protein signals. Changes in temperature have only modest effects on these spectra, given that the DE experiment works well for rigid and dynamic molecules.

A traditional approach in ssNMR is the use of ^1^H‐^13^C cross‐polarization (CP) to detect ^13^C signals (Hartmann and Hahn 1962; Pines et al. 1973). CP transfers polarization from more sensitive nuclei (^1^H) to less sensitive nuclei (e.g., ^13^C, ^15^N), resulting in signal enhancement for the less sensitive (and less abundant) species. This process leverages not only differences in gyromagnetic ratio but also the faster ^1^H relaxation, allowing for faster signal accumulation. However, the efficiency of signal transfer depends on the strength of the dipolar interaction and therefore is significantly affected by motion. In “rigid” molecules, the transfer is highly efficient and fast (sub‐ms transfer times for protonated molecules). However, any motion experienced by a chemical group can lead to dynamic averaging of its dipolar coupling, leading to reduced efficiency of transfer and low(er) signal intensities. In the limit of fast isotropic motion, as is present for small molecules in solution, CP transfer becomes completely ineffective. In the membrane‐cyt c samples (Figure 2c, top) the CP experiment shows a weak signal for the labeled protein, unless the temperature is reduced to control its dynamics. This shows that the protein is highly dynamic in its membrane‐bound state (Kagan et al. 2023; Mandal et al. 2015).

In modern ssNMR, scalar‐based polarization transfer techniques (INEPT‐based) are also popular and powerful, as a complement to the above techniques. These techniques form the basis of liquid‐state NMR, but traditionally were seen as unsuitable for solid samples due to the short ^1^H and ^13^C T_2_ times. Fast T_2_ relaxation leads to signal losses during the INEPT experiment, such that INEPT spectra on dry or solid samples are often devoid of peaks. However, biological (membrane) samples often contain flexible or fluid components that are sufficiently mobile to have longer T_2_ times and give good INEPT‐based spectra (Figure 2c, bottom). A notable consequence of this observation is that one can use INEPT‐based spectroscopy to selectively observe flexible protein segments, as well as the lipids in the liquid crystalline phase (Andronesi et al. 2005; Gross et al. 1995), as illustrated in the shown spectrum (Kagan et al. 2023).

The integrated acquisition and analysis of these simple 1D experiments can shed light on complex features of dynamics and structure in samples. The spectra in Figure 2c illustrate how the three different techniques yield dramatically different spectra when applied to the exact same sample. The DE spectrum gives an overview of the sample composition. The CP spectrum reveals the most rigid parts of the sample, and the INEPT spectrum reveals the highly dynamic or flexible components. Combined, they represent a characteristic fingerprint of the sample in its current state.

### 2.7 Dynamics, temperature dependence, and spectral quality

A deeper understanding of the dynamics can be derived from temperature‐dependent NMR studies. At a certain temperature, the ^1^H NMR shows the freezing of much of the water as a dramatic broadening of the water peak (Figure 2b). Yet, the lipid peaks remain almost unaffected by the same temperature decrease, indicating that they maintain the disordered liquid crystalline phase even below the water freezing point (Mandal and van der Wel 2016). As a result of lowering the temperature, protein signals become more pronounced in the CP spectra, and the lipid signals in the INEPT spectra appear lower in intensity due to reduced molecular motion (Figure 2c). These data shed light on the molecular dynamics of the system but can also inform optimal conditions for performing more time‐consuming 2D and 3D ssNMR spectra. The quality of ^13^C protein spectra can show significant variations with temperature that are dependent on various sample parameters. One key factor is the phase behavior of the lipid membrane, since lipids at high temperature form a fluid liquid crystalline bilayer but at low‐temperature transition to a gel‐like highly ordered structure. Above the lipid membrane melting point (*T*_*m*_), the membrane dynamics can reduce spectral quality due to reductions in (CP) signal intensity and line broadening. Conversely, in a gel phase membrane, the CP signals may increase due to decreased motion, but heterogeneity in the membrane can also lead to line broadening. The extent and origin of the line broadening depends on the type of sample. Membrane‐active peptides are likely more sensitive to the dynamics of surrounding lipids (Su and Hong 2011), than tightly folded larger membrane proteins (Eddy et al. 2012). For instance, the structure of a membrane‐spanning β‐barrel ion channel can be dictated by intra‐protein interactions that make the NMR spectra much less sensitive to the state of the surrounding membrane (Eddy et al. 2012). Other peptides or proteins may be more dependent on interactions with the surrounding membrane, which makes their linewidth sensitive to the gel/liquid transition and can even lead to changes in conformation (Su et al. 2008). For proteins whose dynamics are dictated by those of the membrane, one may find that a particular temperature range (not far from *T*_*m*_) can provide the best compromise of signal intensity and linewidths (Banigan et al. 2015; Li et al. 2019; Mandal et al. 2015). To add further complexity, the lipid and protein dynamics that regulate spectral quality are also sensitive to other sample aspects, notably including the hydration level. Variations in the hydration level between otherwise identical samples can modulate how spectral quality depends on the measurement temperature.

### 2.8 Seeing the lipids by ssNMR

Given the functional importance of the (dynamic) membrane, it is important to note the applicability of ssNMR to also study the lipids (van der Wel 2014). A classic approach uses ^31^P NMR to characterize phospholipid headgroups (Figure 2d–g). ^31^P has a natural abundance of 100% and is highly sensitive. ^31^P MAS NMR spectra can be used to distinguish different phospholipids, while static ssNMR can reveal anisotropic interactions that probe the molecular (re)orientation of the lipid (Seelig 1978; Cullis and de Kruijff 1979; Schiller et al. 2007). An important use of ^31^P NMR is to distinguish different bilayer and non‐bilayer phases (see Figure S2) (Cullis and de Kruijff 1979; van der Wel et al. 2000). Figure 2 shows static ^31^P spectra recorded to assess the CL‐containing liposomes (Figure 2d,e), with and without the protein (Kagan et al. 2023). The spectra on the CL‐containing liposomes are characteristic of a fluid lipid bilayer (red spectra). On the other hand, the inclusion of mono‐lyso‐CL lipids (MLCL) resulted in a more complex lineshape that featured a co‐existing distinct bilayer phase with elevated dynamics (blue spectra) (Powell and Marsh 1985). Analysis of the ^31^P CSA can also be done under (moderate) MAS by analyzing spinning side bands (Figure 2f,g). ^31^P NMR can also distinguish the presence of LUVs or MLVs inside the sample, based on distinct lineshapes (Figure S1d). In some cases, we have observed that samples prepared initially as LUVs (confirmed with the expected ^31^P lineshape) can convert to MLVs (based on ^31^P) during extended ssNMR measurement times (Mandal et al. 2015).

Also, other nuclei are useful in studies of lipids. ^13^C, ^1^H, and ^2^H NMR have been deployed to probe the acyl chains that make up the membrane core. Order parameter measurements are powerful means to probe the (dis)order in the lipid bilayer and determine changes in membrane thickness (Dvinskikh et al. 2005; van der Wel 2014). Here it is worth noting that ^1^H/^13^C NMR is powerful even in samples in which the lipids are not isotopically labeled. ^2^H NMR requires deuterated lipids, but these are commercially available. Notably, there are growing numbers of studies in which ^13^C labeling of lipids is used with good effect (Della Ripa et al. 2018; van Beekveld et al. 2022).

### 2.9 Optimization of conditions for advanced ssNMR

Thus, prior to advanced 2D and 3D ssNMR studies, which are time‐consuming, it is often useful to deploy 1D spectra on test samples to optimize experimental conditions. This includes parameters like hydration level, differences in lipid composition, protein: lipid ratios, and the measurement temperature. This enumeration of parameters illustrates the potential complexity of these types of studies, given that there is a semi‐infinite optimization space. That said, one may identify two key characteristics of obtained spectra: insufficient signal intensity or overly broad signals. Both can be traced to the dynamic properties of the sample that may be modulated through the choice of conditions. High sample dynamics can be attenuated by lower measurement temperatures, but one can also consider using a lower hydration level or more rigid membrane compositions. The latter refers to the fact that lipids with saturated acyl chains tend to be more rigid than those with double bonds. Moreover, membranes with elevated amounts of cholesterol tend to be more rigid than those lacking cholesterol. A consideration in such choices is, however, that certain sample conditions may be considered less physiologically relevant.

### 2.10 2D ^13^C‐ ^13^C spectra to probe residue‐type signatures

**FIGURE 3:**
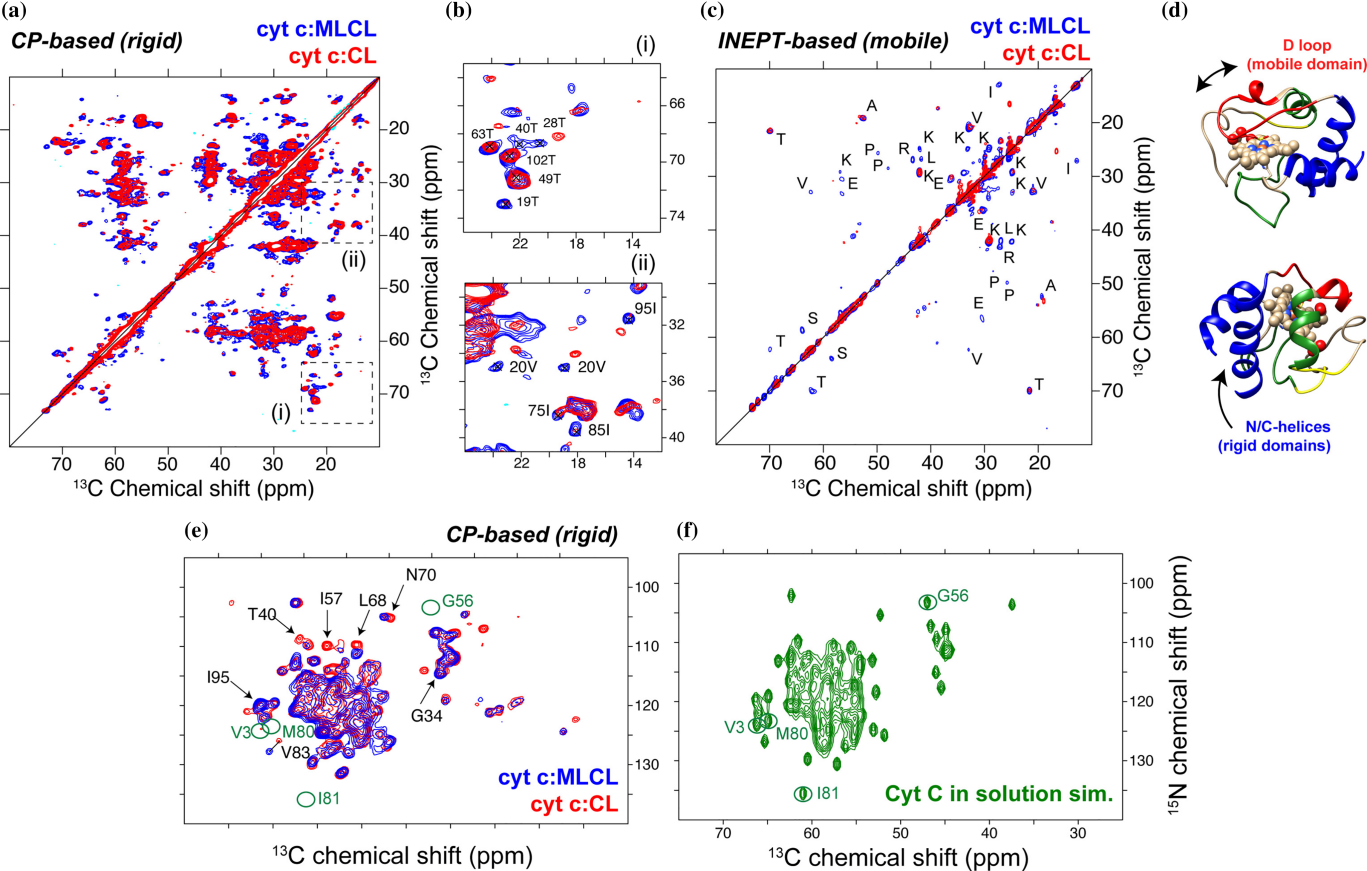
2D MAS NMR of cyt c:ML(CL) complexes and increased dynamics of cyt c *Ω* D loop in the presence of MLCL. (a) Superposition of 2D ^13^C‐^13^C CP‐based DARR NMR spectra of cyt c:MLCL complex (blue) and cyt c:CL complex (red) recorded at 253 K (a). (b) Zoomed in regions. (c) Superposition of 2D ^13^C‐^13^C INEPT‐TOBSY NMR spectra of the same complexes recorded at 278 K. (d) Cartoon representations of cyt c where the *Ω* D loop and the blue foldons are indicated. Figure reprinted with permission from Springer Nature from Kagan et al. (2023).

While 1D spectra can be useful, multidimensional NMR permits more atom‐ or residue‐specific interpretations. 2D ^13^C‐^13^C correlation spectra can often be straightforward to set up and relatively fast to acquire with decent signal‐to‐noise. These types of homonuclear spectra can nonetheless give considerable insight into the behavior of the (labeled) protein, revealing segmental dynamics, secondary structure, and degree of foldedness. A widely used experiment, often deployed as a next step after 1D characterization, is the 2D CP‐based homonuclear ^13^C‐^13^C measurement which performs well at modest MAS rates, based on dipolar‐assisted rotational resonance (DARR) or related methods (Hou et al. 2013). On cyt c bound to vesicles with either CL‐ or MLCL (Figure 3a), high‐quality 2D DARR spectra could be obtained (Kagan et al. 2023; Li et al. 2019; Li et al. 2021). The well‐resolved peak patterns show that the overall protein fold must be intact. If significant protein unfolding had occurred, we would have observed broader peaks and a lower signal‐to‐noise ratio (Li et al. 2021). Figure 3 also shows how the impact of special lipid compositions can be analyzed, overlaying spectra obtained with normal CL and lyso‐CL, with the latter associated with a disease called Barth syndrome. These simple 2D spectra can be a workhorse technique for residue‐specific changes in structure and dynamics, for example, upon substrate binding or changes in lipid composition.

Some parts of membrane‐bound proteins can be too dynamic to be visible in these CP‐based spectra, but are selectively detected in INEPT spectra. J‐based multidimensional methods can be deployed (Andronesi et al. 2005). For instance, in our experiments, we commonly deploy 2D ^13^C‐^13^C TOBSY spectra based on INEPT polarization transfers (Figure 3c). These simple 2D spectra can be sufficient to recognize specific amino acid types, permitting tentative assignments and comparisons to known assignments (from solid state or solution NMR). Here, we saw in the TOBSY spectra residue types that pointed to the protein's important Ω D loop (residues 71–85). Combined, a comparison of 1D and 2D dynamics‐sensitive experiments revealed lipid‐dependent changes, which suggested lipid‐induced changes in motion of specific protein segments. In other work, we also looked at other lipid types beyond CL (Li et al. 2021). The displacement of this Ω D loop is thought to regulate access to the protein's heme cavity, impacting peroxidase activity that underpins its role in apoptosis (and Barth syndrome).

### 2.11 De novo assignments via 2D and 3D (heteronuclear) MAS NMR

**FIGURE 4:**
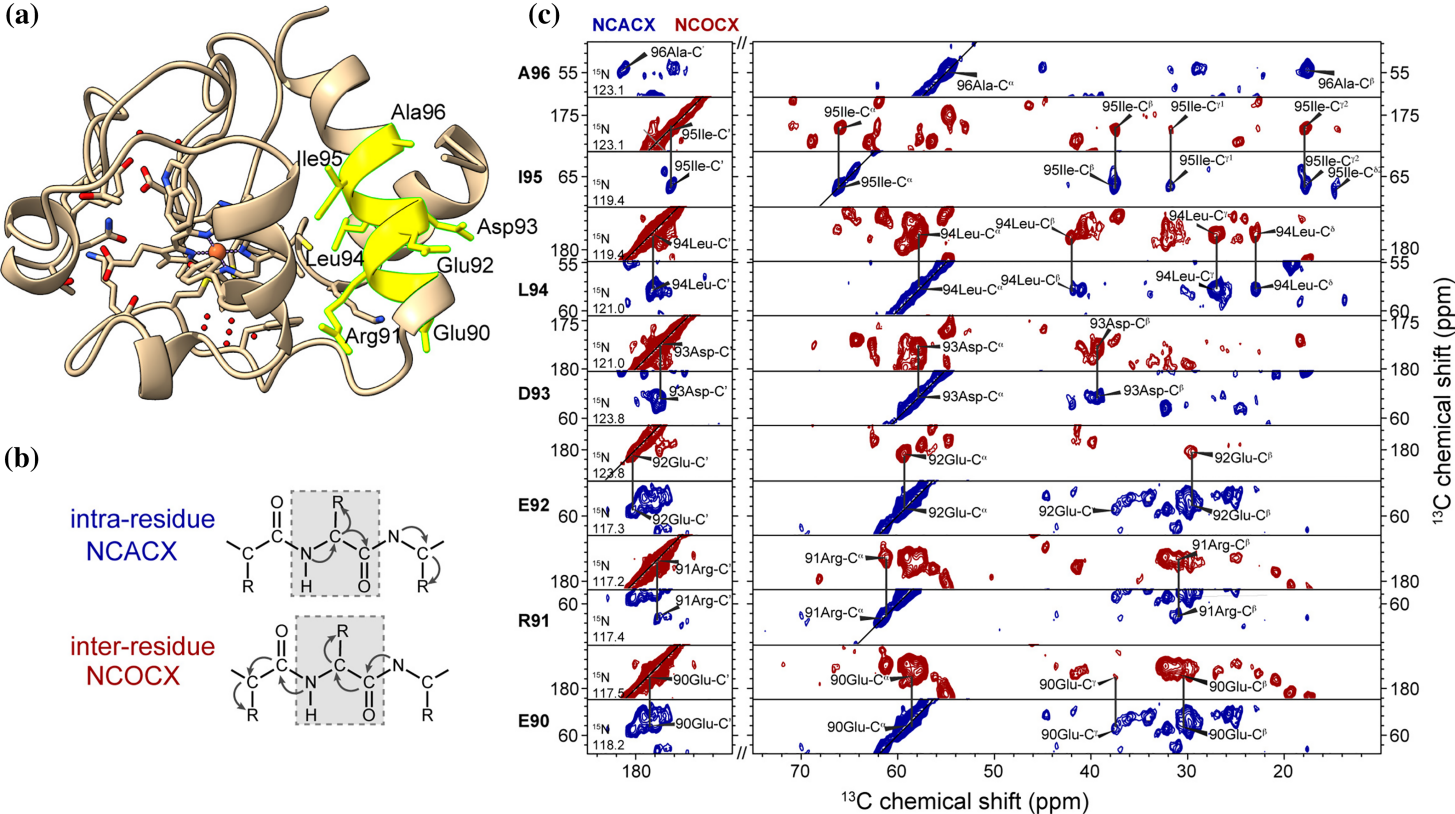
Protein assignments by ssNMR. (a) X‐ray crystal structure of horse heart cyt c (PDB ID 1HRC; Bushnell et al. 1990), with the residues 90–96 indicated in yellow and with text labels. Figure prepared with UCSF ChimeraX software. (b) Schematic descriptions of polarization transfer pathways in NCACX and NCOCX experiments used in ssNMR assignments. (c) Selected 2D slices from 3D NCACX and NCOCX assignment spectra, showing the ^13^C‐^13^C correlations at the ^15^N frequencies (listed bottom left) for residues 90–96. Panel (c) was adapted with permission from Li et al. (2019).

While residue‐type analysis can be useful, along with comparisons to known NMR assignments, further studies often depend on de novo ssNMR assignments. These are usually determined via 2D and 3D spectra featuring ^13^C‐^13^C and ^13^C‐^15^N correlations that establish connectivity between atoms in sequential amino acids (Higman et al. 2009; Shi and Ladizhansky 2012). Previously published (Li et al. 2019) example spectra from such 3D experiments on the membrane‐bound cyt c protein are included in Figure 4. One can map these sequence segments onto the protein's primary sequence to establish complete peak assignments. Ongoing efforts toward software‐supported assignment procedures speed up the process (Higman et al. 2009; Klukowski et al. 2022; Sperling et al. 2010). With assignment data in hand, one can identify the location of secondary structures, based on the fact that residue‐specific chemical shifts can reveal the location of secondary structure elements (Luca et al. 2001; van der Wel 2021). Such analysis can already be useful to validate models derived from homology or AlphaFold modeling. Moreover, partial or complete assignments pave the way for residue‐specific structural and dynamic studies. The assignment experiments are not necessarily difficult to measure, but do take some work in terms of setup and can be time‐consuming to perform and analyze. One important point of “double CP” type experiments (such as those in Figure 4) is that they are notably sensitive to the disrupting effects of motions in the protein and membrane; more so than basic ^13^C‐^13^C 2D spectra (Shi et al. 2009). This can present a challenge for those membrane proteins that are more dynamic than, for example, amyloid fibrils or protein crystals.

### 2.12 Atomic‐level protein structure and dynamics measurements

From a structural biology perspective, the above techniques are merely a precursor to measurements that enable an atomic‐level protein structure to be resolved. The most common approach to solving protein structures by ssNMR depends on measurements of structural constraints: (backbone) dihedral angles along with distance constraints (Comellas and Rienstra 2013). The former are usually derived from the assigned chemical shifts but can also be informed by explicit torsion angle measurements (van der Wel 2021). The distance restraints can be based on ^13^C‐^13^C distances measured by dipolar recoupling methods but can also include ^1^H‐^1^H, ^13^C‐^15^N, and other combinations. The structure‐determination process has been discussed in several prior papers (McDermott 2009; Schneider et al. 2010; Shi and Ladizhansky 2012). To complement structural measurements, one can perform dynamics characterization in a residue‐specific manner, based on a diversity of techniques (Schanda and Ernst 2016). However, comprehensive structural and dynamic analysis is beyond the scope of this report, and we will here focus next on identifying and probing drug or ligand interactions.

### 2.13 Studying ligand–membrane protein interactions by ssNMR

The above methods show the potential of ssNMR in characterizing and fingerprinting the structure and dynamics of proteins in 1D and 2D spectra. Next, we look at how these techniques can be used to analyze the interactions between membrane proteins and drugs or ligands.

#### 2.13.1 Detecting interactions by tracking peak positions and intensities by 2D NMR

**FIGURE 5:**
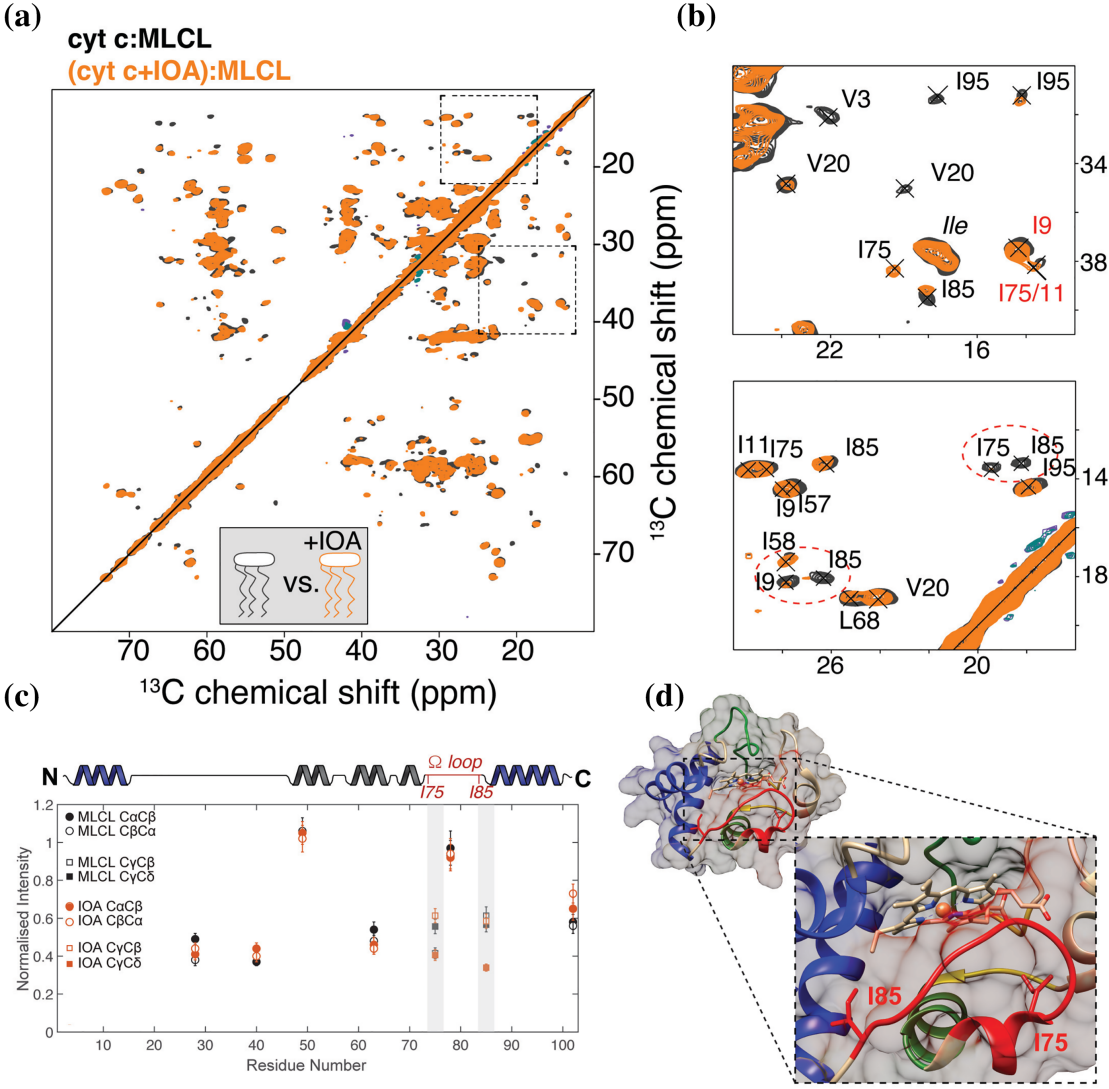
Probing ligand–protein interactions by 2D NMR. (a, b) Superimposition of 2D ^13^C‐^13^C CP‐based DARR NMR spectra (a) and zoomed regions (b) of cyt c bound to DOPC:MLCL (2:1) vesicles in the absence (black) and presence (orange) of 4× excess inhibitor molecule IOA. (b) Dashed ovals mark peaks for Ile75/Ile85 that are diminished in the IOA sample relative to the drug‐free control spectrum. (c) Normalized peak intensity in the absence (black) and presence (orange) of IOA, for Thr Cα/β peaks and Cβ/γ of Ile75 and Ile85. (d) Representation of cyt c, with inset showing Ile75 and Ile85 (in sticks) and the Ω‐loop (red). Figure reprinted with permission from Springer Nature from Kagan et al. (2023).

The most obvious NMR reporter of an interaction is the chemical shift. In solution NMR, chemical shift perturbation (CSP) analysis is a common method to localize the binding site and affinity of a ligand, with changes in the resonances resulting from interaction with the ligand. The underlying principle is that the chemical shift of residues involved in the binding is different in the bound and unbound states. In the slow exchange regime (*k*_ex_ << Δ*ν*) the two conformations (free and bound) appear as separate peaks. In the fast exchange regime (*k*_ex_ >> Δ*ν*), observed resonances move linearly from the free to the bound state, with slight broadening at intermediate ligand concentrations. In intermediate cases (*k*_ex_ ≈ Δ*ν*), peaks disappear to the extent that it becomes difficult to track the binding event. However, translation from solution NMR to ssNMR is complicated by the fact that ssNMR samples are typically studied under conditions of limiting hydration (Mandal et al. 2017). This implies that the concentrations of all sample components are very high, with very limited aqueous volumes for the ligand buffer solution. Additionally, broader lines and peak overlap limit the sensitivity of CSPs in ssNMR. Nonetheless, the determination of CSPs comparing samples with and without ligands is a common first approach. This can be based on the 2D or 3D spectra discussed above, in the presence and absence of a bound ligand. In our example, to follow the binding of an inhibitor to the cyt c‐MLCL complex, we prepared the same cyt c‐MLCL complex with and without the imidazole oleic acid (IOA) compound, an inhibitor of peroxidase activity (Atkinson et al. 2011; Kagan et al. 2023). Spectra were measured with and without the compound, in the form of 2D ^13^C‐^13^C DARR spectra for the two complexes (Figure 5a,b). This allowed the determination of CSPs, but these were rather small and not the most indicative sign of interactions.

A more striking feature in our spectra was a change in the intensities and linewidths of specific peaks. Changes were observed at residues Ile9, Ile75, and Ile85. Interestingly, these residues are in the Ω‐loop D, which defines the heme cavity and was noted to be particularly dynamic above, based on the comparative analysis of 2D INEPT‐ and CP‐based spectra. Our data suggested that the binding of the ligand led to changes in the motion of these residues. On the other hand, the overall protein dynamics remained largely unaffected, at least in terms of changes in the (CP NMR) intensities of Thr peaks throughout the protein (Figure 5c). It is worth noting that a deeper understanding of these dynamics changes would be enabled by systematic measurements of dynamics parameters, based on dipolar order parameters and/or relaxation data (Schanda and Ernst 2016). Thus, one can use ssNMR to probe how an inhibitor can have an impact on residue‐specific dynamics, which here was more obvious than any changes in peak position. The pharmacological importance of (modulating) membrane protein dynamics has been noted in the GPCR research field (Conflitti et al. 2025).

#### 2.13.2 Detecting interactions via dipolar recoupling techniques

**FIGURE 6:**
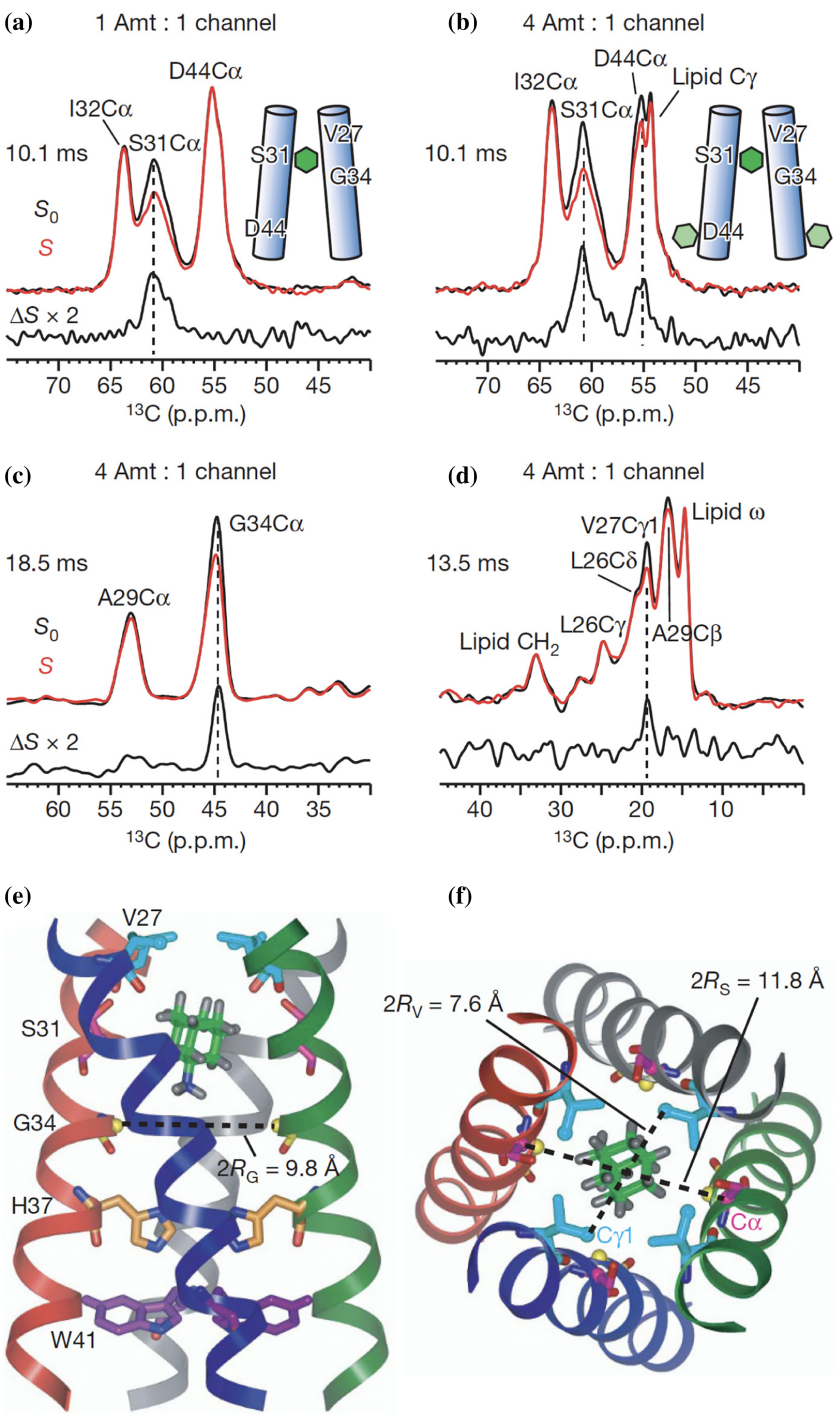
Probing drug–protein interactions with REDOR ssNMR. (a) An example of using ^13^C{^2^H} REDOR to probe drug–protein interactions, from the Hong lab studies of the M2 channel in DMPC membranes (Cady et al. 2010). Control (S0), REDOR dephased (S, red) and difference (ΔS) spectra at specified mixing times were shown. Ser31, Ile32, Asp44‐labeled (SID) M2 at the stoichiometric ratio of amantadine/protein = 1:1. (b) SID–M2 at the fourfold excess ratio of inhibitor. Ser31 Cα was dephased under both conditions but Asp44 Cα was dephased only when Amt was in excess. (c, d) Leu26, Val27, Ala29, and Gly34‐labeled (LVAG) M2 at amantadine/protein = 4:1. (e) Side view showing amantadine in the high‐affinity luminal site of the M2 tetramer. (f) Top view showing the Ser31 and Val27 pore radii. Figure reproduced with permission from Springer Nature from Cady et al. (2010).

Dipolar recoupling techniques (Griffin 1998) can also be applied to the analysis of an interaction between a substrate and (membrane) protein. One of the most famous and powerful recoupling techniques is the rotational‐echo double‐resonance (REDOR) technique (Gullion and Schaefer 1989). This is an experiment designed to measure the dipolar coupling between two different types of nuclei, such as ^15^N and ^13^C. The main principle behind REDOR is the application of rotor‐synchronized radiofrequency pulses that act on the spins of two interacting nuclei and counteract the averaging of dipolar coupling by MAS. A spin echo is recorded on one nucleus of interest (e.g., ^13^C), while a series of 180° pulses is applied to the coupled nucleus (e.g., ^15^N, ^2^H, ^31^P). By increasing the recoupling time and analyzing the extent of REDOR dephasing, one can determine the dipolar coupling strength and internuclear distance, with sub‐nm resolution. A nice illustration relevant to this paper was on the M2 ion channel of the influenza virus, which has been studied by the Hong, Cross, and Griffin groups and others (Figure 6) (Andreas et al. 2010; Andreas et al. 2012; Cady et al. 2009; Cady et al. 2010; Cady and Hong 2008; Can et al. 2012; Hu et al. 2010). M2 forms a tetrameric proton channel that is the target of influenza drugs like amantadine. However, certain M2 mutations have rendered the virus resistant. Multiple ssNMR groups have looked at the M2 channel, its interactions with drugs, and drug resistance mutations (Andreas et al. 2012; Cady et al. 2009; Cady et al. 2010; Can et al. 2012; Hu et al. 2010). These papers reported on various experiments used for detecting drug interactions. Here we would like to focus on one specific approach taken by the Hong lab, based on the REDOR technique (Cady et al. 2010). Binding a ^2^H‐labeled inhibitor to ^13^C‐labeled protein enabled the use of ^13^C‐^2^H REDOR to identify the sites of interaction between ligand and protein and even the determination of distance constraints (Figure 6). A notable feature of the data in the figure was that the authors combined selective residue‐specific labeling such that 1D spectra could be compatible with residue‐specific insights. This approach was also facilitated by the small size of the M2 polypeptide, which was compatible with solid‐phase peptide synthesis that makes single‐residue labeling quite convenient (Figure 6).

### 2.14 Future perspectives

Our focus has been on ^13^C‐focused studies performed at modest MAS rates. There is a large and growing interest in methods that take advantage of faster MAS rates enabled by ever‐smaller MAS rotor sizes. Then, ^1^H‐detection becomes a powerful approach that boosts sensitivity for very small sample sizes (Nishiyama et al. 2023; Stöppler et al. 2018). Depending on the MAS rate, one may study fully protonated samples (near or above 100 kHz) or get useful data with partial deuteration (around 60 kHz MAS). The precise connections between membrane protein dynamics, (fast) MAS rates, and achievable ^1^H linewidths remain under investigation, with a need for further systematic studies. One potential concern to be verified is that membrane samples may be sensitive to high g forces associated with very fast MAS. Those can lead to dehydration of the lipid phase, with detrimental effects for the physiological relevance and sample integrity (Mandal et al. 2017; van der Wel 2014). That said, ^1^H‐detected fast MAS is proving to be an effective technique for membrane protein studies, and it is an area of active methods development. Another technical advance that is impacting membrane protein analysis is the use of dynamic nuclear polarization (DNP) (Barnes et al. 2008). This technique can offer orders of magnitude increases in signal intensity, enabling ssNMR on highly dilute samples, whole‐cell preparations, and also unlabeled protein samples (Kaplan et al. 2015; Sergeyev et al. 2017; Smith et al. 2023). One known limitation of current DNP applications is that they require low to very low temperatures at which dynamics are suppressed. This means that it is less suitable for studies of motional features of (membrane) proteins. These techniques can also be combined with innovative labeling strategies made by advances in molecular biology, along with (^13^C) labeling of lipids for ssNMR (Della Ripa et al. 2018; van Beekveld et al. 2022). There is also a growing interest in ^19^F NMR for studying membrane proteins, such as GPCRs (Didenko et al. 2013; Shcherbakov et al. 2021). This is because of its high sensitivity and responsiveness to local conformational and electronic environments. Combining these developments, ssNMR is increasingly applied to membrane proteins (and peptides) in native membranes and under cellular conditions (Kaplan et al. 2016; Medeiros‐Silva et al. 2018; Ward et al. 2015). Looking beyond NMR per se, there is a clear trend toward integrative approaches, whether through combination with cutting‐edge cryo‐EM or emerging AI‐assisted software tools.

## 3 CONCLUSION

We have summarized how ssNMR can be used to probe membrane proteins in their native environment, and the analysis of interactions with drugs and ligands. The real strength of ssNMR, as illustrated above, is that it offers insights into dynamic systems, and even into dynamic changes induced by ligands and inhibitors. Given that there is a growing awareness of intricate (allosteric) dynamic networks in functional membrane proteins, we anticipate this to continue to be an essential application of ssNMR spectroscopy. It is clear that drugs can act by modulating functional (or dysfunctional) dynamics in membrane proteins, making this an important focal point in advanced drug design.

## AUTHOR CONTRIBUTIONS

**Alessia Lasorsa:** Conceptualization; writing – review and editing; writing – original draft; investigation; visualization; methodology. **Patrick C. A. van der Wel:** Conceptualization; writing – review and editing; funding acquisition; methodology; project administration.

## Supporting information


**Data S1.** Supporting Information.

## Data Availability

The data that support the findings of this study are available from the corresponding author upon reasonable request.
