# Supplementary material for: Solid‐state NMR protocols for unveiling dynamics and (drug) interactions of membrane‐bound proteins
Source: Protein Sci. 2025 Mar 18;34(4):e70102. doi: 10.1002/pro.70102 (PMC11915643; doi:10.1002/pro.70102)
Supplement: Supplementary file 1 — Data S1. Supporting Information. [file PRO-34-e70102-s001.pdf]

Supporting Information

for

Solid-state NMR protocols for unveiling dynamics and (drug) interactions of membrane-bound proteins

Alessia Lasorsa and Patrick C. A. van der Wel*

Contents:

- Figure S1
- Figure S2
- Supplementary Methods
- References cited in the Supplementary Materials

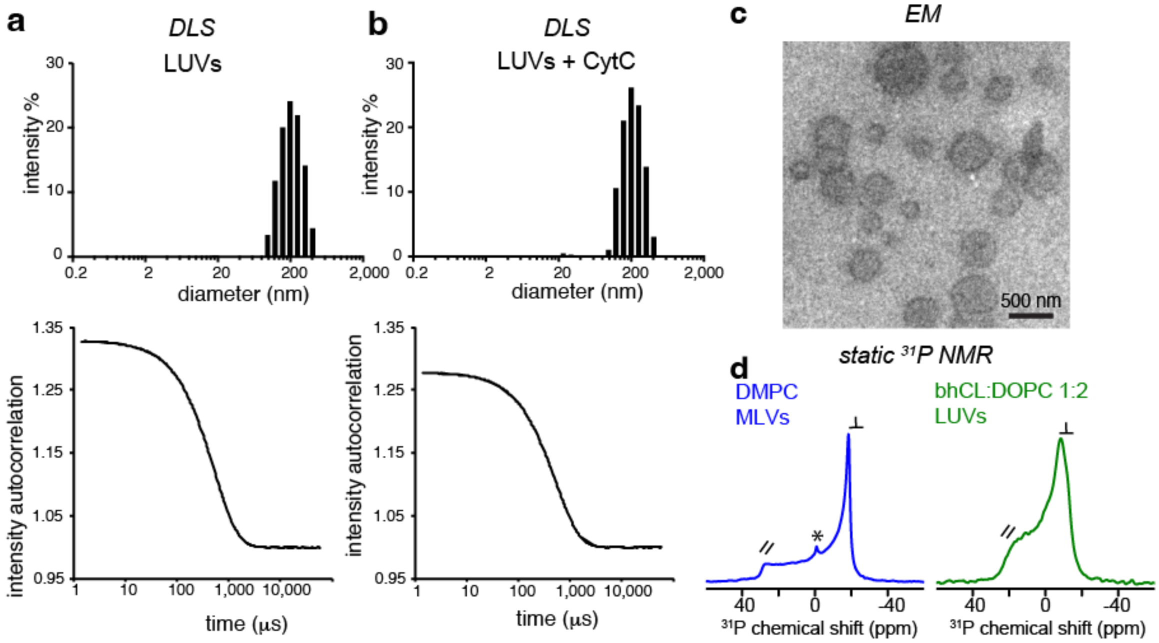

Figure S1. Lipid vesicle analysis. (a,b) Dynamic light scattering (DLS) can be used to analyze the actual LUV diameter. Here, data are shown for LUVs formed by extrusion through a 200-nm pore size membrane, first without bound protein (a), and then with the bound CytC protein (b). The top panels show the interpretated data as a function of the vesicle diameter, whilst the bottom panes show the underlying DLS data. Figure panel adapted with permission from (the SI of) ref. Mandal et al 2015.[1] (c) Negative stain TEM of lipid vesicles can also be used to evaluate their homogeneity and diameter. Here, example data are shown for LUVs prepared from a mixture of PC/CL/cholesterol. These data were adapted with permission from ref. Mandal et al.[2] (d) Static ^{31}P NMR can be used to evaluate the presence of MLVs or LUVs in the sample, based on the differences in the spectra. Shown here are reference data for MLVs prepared from pure DMPC (left), and LUVs prepared from a mixture of bovine heart cardiolipin (bhCL) and DOPC lipids. Both spectra were measured above the lipids' T_m temperature. The larger size, slower vesicle tumbling, and slower lipid diffusion in MLVs result in a larger overall CSA (i.e., distance between the two parallel and perpendicular signals, as marked) and also a 'sharper' appearance (reflecting a longer T_2 relaxation time). The parallel and perpendicular indicators refer to the alignment of the membrane normal relative to the magnetic field[3,4]. The asterisk marks a small amount of isotropic signal (see also Figure S2). See also Supplementary Methods section below.

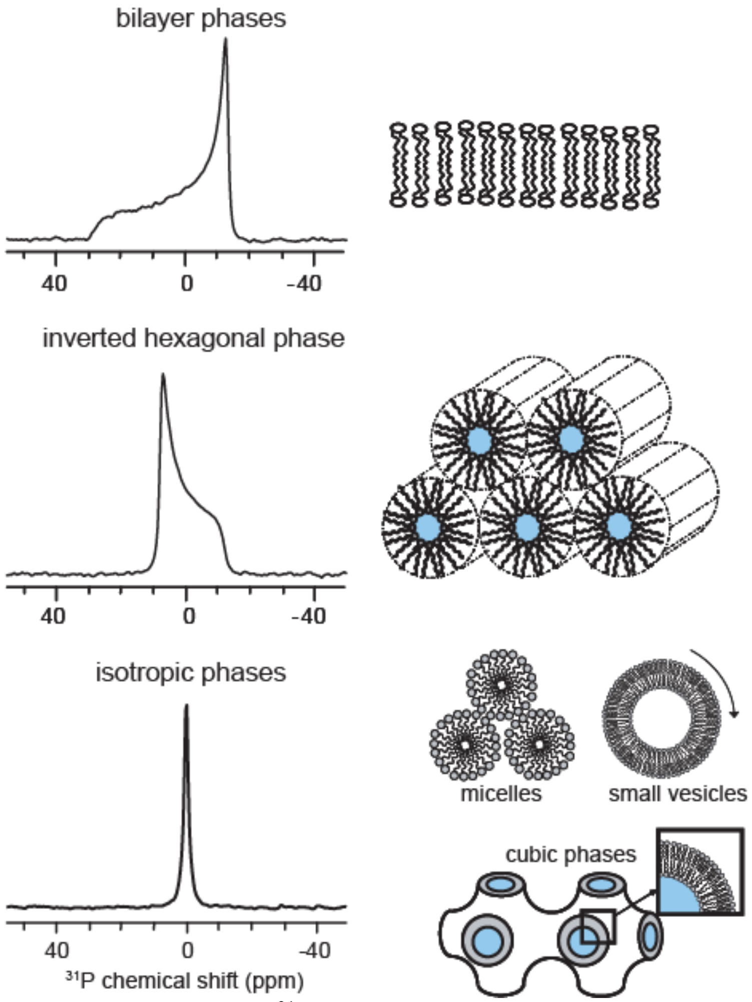

Figure S2. Examples of reference static ^{31}P NMR spectra for different types of phospholipid phases, along with schematic diagrams of each lipid phase type. The hexagonal and cubic phases are often described as 'non-bilayer' phases, and display distinct ^{31}P lineshapes due to characteristic motional averaging of the ^{31}P CSA. Note that isotropic signals can be observed for a variety of different lipid states, and thus cannot always be unequivocally interpreted. For more detailed information on ^{31}P NMR of lipids readers are referred to published works.[5–7]

Supplementary Methods for the ^{31}P NMR data in Figure S1 above.

DMPC MLVs were prepared using 8.5 mg of lipids, initially dissolved in $CHCl_3$ and dried to form a lipid film. The film was then resuspended in 0.5 mL of aqueous buffer (20 mM HEPES, pH 7.4) and subjected to repeated vortexing and freeze-thaw cycles using liquid nitrogen and a 60°C water bath. For bovine heart CL (bhCL):DOPC LUVs (1:2 molar ratio), 4.6 mg of lipids were used, following a similar procedure: the lipid film (evaporated from a $CHCl_3$ solution) was hydrated in 0.2 mL of aqueous buffer (20 mM HEPES, pH 7.4), vortexed, freeze-thawed, and extruded through 400 nm filters. In both cases, samples were packed into rotors via ultracentrifugation, as described in previous work [2]. The static ^{31}P NMR spectra were acquired on a 600 MHz Bruker spectrometer equipped with a 3.2 mm Bruker HX MAS probe. The number of scans was 1k for the first sample and 4k for the second. Additional parameters included a ^{31}P 90° pulse at 60 kHz and ^{1}H decoupling (TPPM) at 50 kHz.

References cited in the Supplementary Materials

1. Mandal A, Hoop CL, DeLucia M, Kodali R, Kagan VE, Ahn J, van der Wel PCA (2015) Structural Changes and Proapoptotic Peroxidase Activity of Cardiolipin-Bound Mitochondrial Cytochrome c. Biophys. J. 109:1873–1884.

2. Mandal A, Boatz JC, Wheeler TB, van der Wel PCA (2017) On the use of ultracentrifugal devices for routine sample preparation in biomolecular magic-angle-spinning NMR. J Biomol NMR 67:165–178.

3. Seelig J (1978) P-31 nuclear magnetic-resonance and head group structure of phospholipids in membranes. Biochim. Biophys. Acta 515:105–140.

4. Van der Wel PCA (2014) Lipid Dynamics and Protein-Lipid Interactions in Integral Membrane Proteins: Insights from Solid-State NMR. eMagRes 3:111–118.

5. Cullis PR, De Kruijff B (1979) Lipid polymorphism and the functional roles of lipids in biological membranes. Biochimica et Biophysica Acta (BBA) - Reviews on Biomembranes 559:399–420.

6. Smith ICP, Ekiel IH Phosphorus-31 NMR of Phospholipids in Membrane. In: Gorenstein D, editor. Phosphorus-31 NMR, Principles and Applications. Orlando: Academic Press; 1984. pp. 447–475.

7. Dufourc EJ, Mayer C, Stohrer J, Althoff G, Kothe G (1992) Dynamics of phosphate head groups in biomembranes. Comprehensive analysis using phosphorus-31 nuclear magnetic resonance lineshape and relaxation time measurements. Biophys. J. 61:42–57.
